# Gender, health and the Sustainable Development Goals

**DOI:** 10.2471/BLT.15.165027

**Published:** 2015-11-01

**Authors:** Veronica Magar

**Affiliations:** a Gender, Equity and Human Rights, World Health Organization, avenue Appia 20, 1211 Geneva 27, Switzerland.

The Sustainable Development Goals (SDG) address, among other global concerns: health and well-being for all (goal 3); gender equality (goal 5); and the reduction of inequality within and among countries (goal 10).^1^ Gender refers to the socially-constructed characteristics of women and men, in all their diversity, while sex refers to purely biological differences. Gender equality is sometimes understood narrowly as concerning only the differences between women and men. To achieve health and well-being for all, a more compelling and nuanced understanding of gender is needed.

To respond to multiple gender inequalities, we need to understand gender as a social and relational process rather than simply emphasizing the difference between women and men. An expanded understanding of gender and health, as shaped by economic, political and cultural relationships,^2^^–^^4^ provides a new starting point for progress on ecologically sustainable development.

In 2012, 7.6% of male deaths were attributed to alcohol, compared to 4.0% of female deaths.^5^ More men than women die of tuberculosis;^6^ in road crashes^7^ and from other violent causes of death.^6^ Social norms and expectations can increase men’s health risks and reduce health-seeking behaviour. Many communities view taking action on health as unmanly, reducing men’s willingness to seek health services.^3^ Masculinity can be expressed in harmful ways, such as violence against women or sexual practices that expose partners to human immunodeficiency virus infection.^8^^,^^9^ Such behaviour can be associated with established social norms of masculinity, but also with the partial breakdown of men’s position in the gender order, under pressures of poverty and economic change.^2^

Gender must be understood within a complex and specific local context. By capturing the different experiences of men and women, gender can be understood as dynamic and layered with a range of multiple, intersecting social determinants that impact on health.

For example, consider the behaviour of two women from the same village. One walks straight through the village, arriving at the health centre for an appointment in twenty minutes. Another walks for two hours around the perimeter of the village to reach the same health centre. The first woman is upper caste and married to the village chief; the second is tribal, and her husband has untreated tuberculosis, partly because of a lack of resources and partly due to gendered biases that make men reluctant to seek medical care. Different experiences of oppression and privilege shape the health, well-being and illness of these villagers. Ethnicity and poverty are determinants^10^ that intersect with gender and together contribute to a longer trip to the health centre. In this respect, the tribal woman may have more in common with her husband, with whom she shares the same communal struggles, than she does with the chief’s wife.

Taken as a whole, the SDGs^1^ reflect an expanded interpretation of gender that includes a range of inequalities and considers men as well as women. Gender equity is grounded in human rights principles and centred on the concept of universality.

Mainstreaming gender requires concrete actions to eliminate inequalities in our policies and practices. We have to ensure that universal health coverage and financial protection measures include those who are most marginalized. We need to monitor health inequalities, collecting data that reflects income, gender, age, race, ethnicity, migratory status, disabilities and where people live.^11^ We must improve the coverage of health services by removing barriers across sectors. Communities can be mobilized through accountability mechanisms^12^ and participatory and action learning groups.^13^ Taken together, such measures will contribute to meeting the SDGs.

It is time to build upon hard-won accomplishments of gender and women’s health with an expanded social justice perspective. This nuanced exploration of gender represents both our biggest challenge and deepest hope for health, well-being and dignity for all.
